# Prevalence of Dental Erosive Wear and Possible Risk Factors among Adolescents and Adults in Poland – A National Survey

**DOI:** 10.3290/j.ohpd.b5656322

**Published:** 2024-08-06

**Authors:** Ewa Rusyan, Izabela Strużycka, Adrian Lussi, Ewa Grabowska, Agnieszka Mielczarek

**Affiliations:** a Dentist, Department of Conservative Dentistry, Warsaw Medical University, Warsaw, Poland. Study design, data collection, manuscript preparation.; b Professor, Department of Comprehensive Dental Care, Warsaw Medical University, Warsaw Medical University, Warsaw, Poland. Study design, data collection.; c Professor, Department of Preventive, Restorative and Pediatric Dentistry, University of Bern, Bern, Switzerland. Study design, manuscript preparation.; d Dentist, Department of Periodontology And Oral Mucosa Diseases, Warsaw Medical University, Warsaw. Statistical analysis.; e Professor, Department of Conservative Dentistry, Warsaw Medical University, Warsaw, Poland. data collection, manuscript preparation.

**Keywords:** BEWE, dental erosion, epidemiological study, risk factors

## Abstract

**Purpose:**

To investigate the prevalence and severity of erosive tooth wear (ETW) and evaluate the determinants of ETW among adolescents and adults in Poland.

**Materials and Methods:**

The study covered three age groups of patients: 15 years old, 18 years old, and adults aged 35-44 years. Calibrated examiners measured ETW according to the Basic Erosive Wear Examination (BEWE) scoring system in 6091 patients. The clinical examination of patients was preceded by a socio-medical study based on a questionnaire consisting of items identifying potential risk factors for ETW.

**Results:**

In all age groups, erosive lesions were most common in the form of initial enamel damage; more advanced lesions (BEWE 2 and 3) were rarely observed among 15-year-olds, while in the group of older adolescents and adults, the percentages were 13% and 20%, respectively. Acidic diet, gender, level of education, and medical conditions were statistically significantly associated with ETW in the examined population. The analysis showed that, depending on age, multiple and statistically significant risk factors for ETW become most apparent in the 35-44 age group, especially with regard to general health. This suggests that the long-term impact of factors and their cumulative effects are critical to the development of ETW.

**Conclusions:**

This is the first large, representative study of ETW in Central and Eastern Europe among adolescents and adults, which indicates the relatively rare occurrence and severity of erosive lesions. The present findings support other longitudinal studies supporting the use of the BEWE system as a valuable standard for assessing erosive lesions and related risk factors among different populations at different ages.

A program for monitoring the oral health of the Polish population has been conducted in cooperation with the Ministry of Health and the Medical University of Warsaw since 1997, with an interruption due to the Covid-19 pandemic. The program aims to assess the current health status of the population and monitor ongoing changes and potential conditions affecting oral health. Following the principles of oral health monitoring outlined by the World Health Organization (WHO), specific age groups are targeted in subsequent years for periodically repeated epidemiological surveys. In addition to assessing caries and periodontal disease, studies conducted between 2011 and 2021 also included the evaluation of tooth erosive wear using the Basic Erosive Wear Scoring System (BEWE). The results obtained are regularly published in the form of studies and recommendations for the Polish healthcare system and as scientific publications.^27–29,32,38,39^

Numerous studies indicate that the prevalence of erosive tooth wear (ETW) is high and has shown an increasing trend in recent years.^18,22,34^ Still, the results obtained show considerable variation and are difficult to compare, mainly due to non-uniform scoring systems, different age groups, sample sizes or nationalities studied. Experts emphasise the marked trend toward greater severity and faster progression of lesions, especially in younger age groups.^11,26,41^ Conducting studies among children and adolescents usually poses fewer recruitment problems compared to adults. The studies conducted in Poland included people aged 35-44 in addition to adolescents. This represents an interesting aspect of the project as it provides insight into the oral health status and health-seeking behaviour of a population with established attitudes. Additionally, it increases the data on the epidemiology of ETW in Central and Eastern Europe, which is otherwise scarce. All study groups were evaluated under standardised clinical and socio-medical study conditions, which provided an opportunity for comparative analysis of potential risk factors for ETW in a relatively homogeneous population in terms of geography, ethnicity, and culture.

The purpose of this study was to assess the prevalence and severity of erosive tooth wear and evaluate the determinants of this type of lesion among adolescents and adults in Poland.

## MATERIALS AND METHODS

### Study Population

The study was part of a nationwide epidemiological survey, performed annually on different age groups as a cross-sectional study. This dental health monitoring program, carried out since 1997, is not a longitudinal study of the same individuals. Instead, a new population sample is randomly drawn every year from a specific age group. Different index age groups are chosen every year, according to the perceived gaps in knowledge regarding the oral health of particular age groups.

The analysis presented in this paper is based on groups examined in 2011 (2639 adolescents 15 years old), 2015 (1869 adolescents 18 years old), and 2021 (1583 adults 35 to 44 years old). Population samples were selected for each of these analyses by multilayer random sampling. In each voivodship, random counties, municipalities, then cities and villages were drawn. Regarding schoolchildren, public schools located in the area were included in the draw. The most common problems in recruiting schoolchildren for the study were: lack of consent from school administration, lack of consent from parents/legal guardians and the eligible adolescents themselves, as well as absence from school on the day of the study. Regarding adults in the randomly selected cities and villages, after prior announcements by local government authorities, social media, religious communities, and health centres, the surveys were conducted among those who reported to designated dental offices on the day of the survey, or at workplaces with dental offices that met the survey requirements. Recruitment problems in the adult population were due to reluctance to provide the socio-medical data, especially income, included in the survey.

### Data Collection

All procedures performed in studies involving human participants were in accordance with the ethical standards of the institutional and/or national research committee and with the 1964 Helsinki declaration and its later amendments or comparable ethical standards. Informed consent was obtained from all individual participants included in the study.

All patients qualified for the study were identified anonymously in the database on the basis of the number assigned, the dentist’s number and the voivodship in which the study was conducted. Inclusion and exclusion criteria were the same for all groups of study subjects except for consent, which was signed by parents or legal guardians on behalf of minors. Excluded from the study were subjects undergoing orthodontic treatment with fixed appliances, subjects bearing fixed prosthetic restorations, and patients without at least one remaining tooth in each of the tested sextants.^2,9^

The study was carried out by dentists-epidemiologists who were first calibrated before each annual survey. Importantly, for the duration of the study, the team of people involved in the project underwent only minor changes, e.g., due to illness or retirement. The vast majority of people, including the doctors performing the calibration, remained the same. All tooth surfaces were examined for erosive wear based on the criteria of the “Basic Erosive Wear Examination” indicator (BEWE Code 0: no erosive tooth wear, Code 1: initial loss of enamel surface, Code 2: distinct defect, hard tissue loss less than 50% of the surface, Code 3: hard tissue lost more than 50% of the surface).^9^ The reliability of the clinical diagnosis was verified in a repeat dental examination, which included a randomly selected 10% of the study sample. A level of agreement of at least 85% (Kappa Statistics) was obtained for the registration of ETW in all surveys.

The calibration consisted of a lecture discussing the BEWE system, presentation of clinical case photos of patients with ETW of varying severity, and presentation of plaster models. This was followed by clinical trials. In the following years there were: 20 subjects 15-16 years of age, 20 subjects 17-21 years of age, and 26 adult patients, 29-46 years old, with and without ETW. All subjects were examined using the BEWE index. Researchers who were uncertain about the severity of the lesions were asked to mark a lower score.

The clinical examination of patients was preceded by a socio-medical study based on a questionnaire consisting of questions identifying potential risk factors for ETW. This paper presents a standardised version of the questionnaire, i.e., only the questions appearing in the same form for all the age groups were analysed in subsequent studies. The items included in the questionnaire concerned demographic data (age, gender and place of residence), factors potentially influencing the development of ETW, i.e., diseases, allergy, asthma, reflux, eating disorders, hygiene and dietary habits. The latter two comprised frequency of toothbrushing, type of brush used, frequency of consumption of acidic drinks and foods, and respondents’ level of knowledge relating to the aetiology of ETW. Table 1 shows the characteristics of the study group.

**Table 1:** Characteristics of the study groups

		15-year-olds	18-year-olds	35– to 44-year-olds	Comparison (χ^2^ test)
Group size		2639 subjects	1869 subjects	1583 subjects	-
Gender	Boys/Men	1271 (48.2%)	922 (49.3%)	617 (39.0%)	p < 0.0001
	Girls/Women	1368 (51.8%)	947 (50.7%)	966 (61.0%)	
Place of residence	Urban	1542 (58.4%)	1033 (55.3%)	887 (56.0%)	p = 0.0812
	Rural	1097 (41.6%)	836 (44.7%)	696 (44.0%)	
Educational level of the respondent or their mother’s (in the case of teenagers)	Primary education	886 (37.2%)	747 (41.8%)	109 (6.9%)	p < 0.0001
	Secondary education	938 (39.4%)	602 (33.7%)	620 (39.2%)	
	Higher education	556 (23.4%)	440 (24.6%)	854 (54.0%)	
General health	Respondent sees themself as generally healthy	2192 85.1%	1601 88.0%	1255 79.3%	p < 0.0001
	Reflux	475 18.4%	24 1.3%	50 3.2%	p < 0.0001
	Eating disorders	95 3.7%	29 1.6%	6 0.4%	p < 0.0001
	Allergies	20 0.8%	184 9.8%	84 5.3%	p < 0.0001
	Asthma	11 0.4%	55 2.9%	36 2.3%	p < 0.0001
Percentage of respondents aware that excessive consumption of fruits and fruit juices dissolves tooth surfaces		322 12.8%	404 22.1%	1055 66.6%	p < 0.0001

The study was approved by the Bioethics Committee of the Medical University of Warsaw.

### Statistical Analysis

Data analyses were performed using PQStat software v. 1.6.8. (PQStat; Poznan, Poland). Descriptive statistics were presented as the number and percentage of participants. The χ^2^ test with Fisher’s post-hoc analysis was used for comparisons of categorical variables, while Mann-Whitney U-test or Kruskal-Wallis ANOVA with Dunn-Bonferroni post-hoc analysis were applied when comparing the quantitative variables between two or more groups, respectively. Spearman’s rank correlation was used to analyse the correlation between quantitative variables. Backward multiple regression was then applied to select the statistically significant variables for the explanatory modelling. p < 0.05 was considered statistically significant in all the analyses.

## RESULTS

### Prevalence of Erosive Tooth Wear in the Study Groups

The percentage of subjects with signs of erosive tooth wear in the study population is shown in Table 2. In all age groups, erosive lesions were most common in the form of initial enamel lesions; more advanced lesions (BEWE 2 and 3) were rarely observed among 15-year-olds, while in the group of older adolescents and adults, the percentages were 13% and 20%, respectively. A clinical study showed that the number of sextants affected also increases very significantly with age – in the group of 35- to 44-year-olds, almost one in five had signs of ETW in all examined sextants.

**Table 2:** Maximum values of the BEWE index and the number of sextants affected by ETW in subjects aged 15, 18 and 35-44 years

Age group	Maximum BEWE (number and % of participants)				Correlation with age (Spearman’s monotonic correlation test)	Number of sextants affected by ETW (number and % of participants)							Correlation with age (Spearman’s monotonic correlation test)
	BEWE = 0	BEWE = 1	BEWE = 2	BEWE = 3		0	1	2	3	4	5	6	
15	1997 75.7%	563 21.3%	74 2.8%	5 0.2%	r = 0.2809 p < 0.0001	1997 75.7%	269 10.2%	177 6.7%	43 1.6%	41 1.6%	15 0.6%	97 3.7%	r = 0.2809 p < 0.0001
	2560 97.0%		79 3.0%										
18	1078 57.7%	540 28.9%	224 12.0%	27 1.4%		1076 57.7%	230 12.3%	235 12.6%	62 3.3%	61 3.3%	32 1.7%	173 9.3%	
	1618 86.6%		251 13.4%										
35-44	722 45.6%	537 33.9%	271 17.1%	53 3.3%		722 45.6%	175 11.1%	163 10.3%	73 4.6%	82 5.2%	69 4.4%	299 18.9%	
	1259 79.5%		324 20.5%										

The analysis of the relationship between the prevalence of ETW and social factors yielded inconclusive results (Table 3). Neither gender nor place of residence were determinants of the prevalence of ETW in any of the age groups. In the group of 18-year-olds, erosive lesions were more common among males and those living in the city. Perhaps due to the relatively short period of time since the eruption of the permanent teeth, no such correlation was observed in the 15-year-olds. In addition, the prevalence of lesions was low in this group. In contrast, among adult subjects, the prevalence of ETW was found to be almost equal regardless of sex and place of residence. However, a correlation was observed between education level and the prevalence of ETW in this group. As the level of education increased, the degree of ETW decreased statistically significantly, although, as further analysis showed, the level of awareness of an erosive diet did not show a correlation with the prevalence and severity of ETW.

**Table 3:** The relationship between the prevalence of ETW and social factors and health

Metrics		Percentage of subjects affected by ETW					
		15	Comparison (χ^2^ test)	18	Comparison (χ^2^ test)	35–44	Comparison (χ^2^ test)
Gender	Male	333/1271 26.2%	p = 0.0328	421/922 45.7%	p = 0.0043	348/617 56.4%	p = 0.2144
	Female	309/1368 22.6%		370/947 39.1%		513/966 53.1%	
Place of residence	Urban	365/1542 23.7%	p = 0.3575	459/1033 44.4%	p = 0.0429	480/887 54.1%	p = 0.8389
	Rural	277/1097 25.3%		332/836 39.7%		381/696 54.7%	
Educational level of the respondent or their mother’s (in the case of teenagers)	Primary	223/886 25.2%	p = 0.4739	316/747 42.3%	p = 0.4102	67/109 61.5%	p = 0.0497
	Secondary	237/938 25.3%		241/602 40.0%		352/620 56.8%	
	Higher	126/556 22.7%		194/440 44.1%		442/854 51.8%	
Respondent sees themself as generally healthy	yes	554/2192 25.3%	p = 0.1191	666/1601 41.6%	p = 0.4162	666/1255 53.1%	p = 0.0388
	no	83/385 21.6%		97/218 44.5%		195/328 59.5%	
Reflux	yes	113/475 23.8%	p = 0.6032	10/24 41.7%	p = 0.9478	35/50 70.0%	p = 0.0243
	no	524/2102 24.9%		781/1845 42.3%		826/1533 53.9%	
Eating disorders	yes	18/95 18.9%	p = 0.1839	12/29 41.4%	p = 0.9175	6/6 100%	p = 0.0246
	no	619/2482 24.9%		779/1840 42.3%		855/1577 54.2%	
Allergies	yes	4/20 20.0%	p = 0.6234	75/184 40.8%	p = 0.6517	51/84 60.7%	p = 0.2318
	no	633/2557 24.8%		716/1685 42.5%		810/1499 54.0%	
Asthma	yes	1/11 9.1%	p = 0.2285	29/55 52.7%	p = 0.1129	26/36 72.2%	p = 0.0298
	no	636/2566 24.8%		762/1814 42.0%		835/1547 54.0%	

Interesting results were obtained with regard to systemic diseases, proving the correlations with ETW prevalence documented before by other authors. Subjects with eating disorders, gastroesophageal reflux disease and asthma presented a higher prevalence of ETW. At the same time, this correlation occurred only in the group of adults aged 35-44.

### Diet

The dietary preferences of the subjects were determined based on the results of the questionnaire, in which respondents were asked to mark the frequency of consumption of the listed acidic foods. Overall, the consumption of acidic foods and drinks was comparable in all age groups. However, their nature changed. Teenagers were more likely to consume acidic drinks, such as fruit teas, fruit juices, isotonic drinks, energy drinks and other sweetened beverages. Subjects aged 35-44, on the other hand, were more likely to consume pickles and hot sauces, as well as fruits (Tables 4 and 5).

**Table 4:** The relationship between the prevalence of ETW and diet

	Consumption at least once a day – number and percentage of subjects			Comparison (χ^2^ test with Fisher’s post-hoc analysis)	Consumption several times a day – number and percentage of subjects			Comparison (χ^2^ test with Fisher’s post-hoc analysis)
Age group	15	18	35–44		15	18	35–44	
fruit	1958 79.6%	1360 75.8%	1365 86.2%	p = 0.0053 Less often among the group of 18-year-olds, more often among 35-44-year-olds	471 19.1%	285 15.9%	313 19.8%	p = 0.0053 Less often among the group of 18-year-olds
juices	1702 69.2%	1178 65.7%	1026 64.8%	p = 0.0067 more often in the group of 15-year-olds	662 26.9%	408 22.7%	183 11.6%	p < 0.0001 frequency decreasing with age
Fruit teas	1318 53.6%	954 53.3%	764 48.3%	p = 0.0018 less frequently among 35-44-year-olds	402 16.3%	320 17.9%	230 14.5%	p = 0.0312 more often among the group of 18-year-olds, less often among 35-44-year-olds
Isotonic drinks	282 11.5%	257 14.4%	219 13.8%	p = 0.0120 less frequently in the group of 15-year-olds	115 4.7%	85 4.8%	28 1.8%	p < 0.0001 less often among 35-44-year-olds
Sweetened beverages	996 40.5%	676 37.7%	578 36.5%	p = 0.0255 frequency decreasing with age	336 13.7%	236 13.1%	120 7.6%	p < 0.0001 less often among 35-44-year-olds
Energy drinks	225 9.2%	228 12.7%	226 14.3%	p < 0.0001 less frequently in the group of 15-year-olds	91 3.7%	74 4.1%	23 1.5%	p < 0.0001 less often among 35-44-year-olds
Pickles, hot sauces	128 5.2%	133 7.4%	550 34.7%	p < 0.0001 frequency increasing with age	43 1.8%	38 2.1%	21 1.3%	p = 0.2105

**Table 5:** Correlation between consumption of acidic foods and drinks and severity of ETW

	Typical frequency of consumption				Correlation with total ETW severity (Spearman coefficient)		
Age group	15	18	35–44	Comparison (Kruskal-Wallis ANOVA with post-hoc analysis by Dunn with Bonferroni correction)	15	18	35–44
Fruit	daily	daily	daily	p < 0.0001 more often among 35-44-year-olds, less often among the group of 18-year-olds,	r = 0.0192 p = 0.3414	r = 0.0107 p = 0.6496	r = -0.0027 p = 0.9156
Juices	daily	daily	daily	p < 0.0001 frequency decreasing with age	r = 0.0138 p = 0.4953	r = -0.0059 p = 0.8036	r = 0.0692 p = 0.0058
Fruit teas	daily	daily	Several times a month	p < 0.0001 less often among 35-44-year-olds	r = -0.0195 p = 0.3331	r = 0.0572 p = 0.0156	r = 0.0412 p = 0.1009
Isotonic drinks	occasionally	occasionally	occasionally	p = 0.0001 less often among 35-44-year-olds	r = 0.0367 p = 0.0694	r = 0.0335 p = 0.1573	r = 0.0423 p = 0.0927
Sweetened beverages	Several times a month	Several times a month	Several times a month	p < 0.0001 frequency decreasing with age	r = 0.0436 p = 0.0308	r = 0.0806 p = 0.0006	r = 0.0498 p = 0.0476
Energy drinks	occasionally	occasionally	occasionally	p = 0.0006 less often among 35-44-year-olds	r = 0.0356 p = 0.0774	r = 0.0018 p = 0.9394	r = 0.0171 p = 0.4971
Pickles, hot sauces	occasionally	occasionally	Several times a month	p < 0.0001 frequency increasing with age	r = 0.0258 p = 0.2021	r = 0.0114 p = 0.6305	r = -0.0522 p = 0.0376
Total consumption of acidic foods and beverages (on a scale of 7 to 28): median Q1-Q3 min-max	14 13–16 7–28	14 12–16 7–28	14 13–17 7–28	p = 0.1038	r = 0.0332 p = 0.0995	r = 0.0572 p = 0.0153	r = 0.0546 p = 0.0298

In the adult group, there was no correlation between BEWE and declared alcohol consumption; 44.2% of respondents reported that they had not consumed alcohol in the past month, 50.3% that they had consumed less than 1 drink per day, and only 4.5% 1 drink per day, while 15 people reported more than 1 drink per day. The declared low consumption frequency and the type of alcohol (mainly beer and spirits, rarely wine) also explains, at least in part, the lack of effect of alcohol on ETW in the adult population. In the young adult group, few respondents gave a positive answer to the questions on alcohol and drug use, with the frequency of use being sporadic. Therefore, no relationship was found between these variables and the BEWE index.

Statistical analysis showed that the maximum severity of ETW correlated – especially in the older groups, among 18- and 35- to 44-year-olds – with total exposure to acidic drinks and foods. In addition, sweetened beverages such as Coca-Cola®, Pepsi® and orangeade were the most common products correlated with erosive lesions. Surprisingly, a negative correlation with the consumption of pickles was found in the 35- to 44-year-old group. There is a suspicion, which cannot be confirmed on the basis of the findings, that people affected by erosive tooth wear avoid very acidic foods due to discomfort, caused for example by tooth sensitivity. All correlations, even if statistically significant, were very weak – r coefficient ρ (-0.1; 0.1).

The awareness of the harmful effects of acids from fruits and juices increased very markedly with age. At the same time, while in 15-year-olds, who had little health awareness, the level of knowledge did not correlate in any way with the products consumed. In both of the older groups, greater awareness correlated with greater consumption of fruits, pickles, hot sauces and lesser consumption of sweetened and energy drinks (Table 6). It can be assumed that individuals who were more aware of the harmful effects of an acidic diet presented an overall greater health awareness and chose healthier foods, such as fruits, despite their erosive potential, but likely based on other prevailing health benefits.

**Table 6:** Correlation between consumption of acidic foods and drinks and awareness of their harmful effects on teeth

	Comparison of consumption of acidic foods and drinks according to the answer to the question Do you agree with the statement? Excessive consumption of fruits and fruit juices dissolves tooth surfaces (Mann-Whitney U-test)		
	15	18	35–44
Fruits	p = 0.7224	p = 0.0013 (more often among subjects aware of the side effects of juices and fruits)	p < 0.0001 (more often among subjects aware of the side effects of juices and fruits)
Juices	p = 0.6357	p = 0.2410	p = 0.6294
Fruit teas	p = 0.6543	p = 0.3745	p = 0.6106
Isotonic drinks	p = 0.6715	p = 0.4233	p = 0.4007
Sweetened beverages	p = 0.2779	p = 0.0098 (less often among subjects aware of the side effects of juices and fruits)	p = 0.0103 (less often among subjects aware of the side effects of juices and fruits)
Energy drinks	p = 0.8315	p = 0.1623	p = 0.0002 (less often among subjects aware of the side effects of juices and fruits)
Pickles, hot sauces	p = 0.2584	p < 0.0001 (more often among subjects aware of the side effects of juices and fruits)	p = 0.0057 (less often among subjects aware of the side effects of juices and fruits)
Acidic foods and drinks in total	p = 0.8203	p = 0.9872	p = 0.8979

### Multivariate Analysis

Multivariate analysis was conducted taking the following into account: as the dependent variable: maximum BEWE; as independent variables: age group, gender, place of residence (urban/rural), mother’s level of education/own level of education, systemic diseases (asthma, reflux, allergies, eating disorders), theoretical knowledge about erosion and preferred diet.

The analysis was conducted using the backward multiple regression method until only factors with p < 0.05 remained in the model. The analysis was then repeated separately for each age group. The resulting models are shown in Table 7.

**Table 7:** Multivariate analysis of variables correlating with the severity of ETW

Analysis for the entire study population. Dependent variable: maximum value of BEWE		
**Independent variables**	**p-value**	**Standardised β coefficient**
Age	< 0.0001	0.27
Female gender	0.0001	-0.07
Education / Mother’s education	0.0002	-0.05
Allergies	0.0005	0.04
Diet	0.0452	0.03
Analysis for the group of 15-year-olds. Dependent variable: maximum value of BEWE.		
**Independent variables**	**p-value**	**Standardised β coefficient**
Female gender	0.0096	-0.05
Analysis for the group of 18-year-olds. Dependent variable: maximum value of BEWE.		
**Independent variables**	**p-value**	**Standardised β coefficient**
Female gender	0.0003	-0.08
Theoretical knowledge	0.0418	-0.05
Analysis for the group of 35-44-year-olds. Dependent variable: maximum value of BEWE		
**Independent variables**	**p-value**	**Standardised β coefficient**
Subject’s education	< 0.0001	-0.13
Female gender	0.0011	-0.08
Reflux	0.0045	0.07
Eating disorders	0.0100	0.06

The most important factor correlating with ETW was the age of the subjects (β = 0.27). In the combined analysis of all three groups, less significant factors were gender (lower frequency in girls, β = -0.07), education (the higher the level, the lower the severity of erosive lesions, β = -0.05), allergy (β = 0.04) and overall consumption of acidic drinks and foods (β = 0.03).

When analysed by age, numerous risk factors for ETW were only statistically significant among adults. Among adolescents, virtually the only such factor was gender (lower severity of erosive lesions in girls, β = -0.05 in 15-year-olds, β = -0.08 in 18-year-olds), while in 18-year-olds, the awareness of tooth erosion wear and its causes was an additional protective factor (β = -0.05).

## DISCUSSION

The most surprising result of the present study, compared to other authors, especially those in Europe, is the relatively low percentage of subjects with ETW, especially the more advanced forms (BEWE 2 and 3) in all age groups studied.^6,10,21,31,35,42^ The prevalence and severity of the lesions increased with the age of the subjects. The observed progression is in line with studies conducted by Hasselkvist et al^20^ and many other authors.^1,3,13,17,26^ Unfortunately, due to the character of the study, no longitudinal observations in the same groups of participants could be performed. The study was carried out over the years in different rural and urban areas, although in the same provinces, throughout the country. Comparing the data obtained in 2001 for 12-year-old children in Wroclaw (Poland) to the present results for 15-year-old adolescents, an unfavourable upward trend can be noted (BEWE 8.3% vs 24.3%).^24^ Also, a 2018 study among young adults in Lviv (Ukraine) and Lublin (Poland) showed a higher percentage of those affected by erosive tooth wear, 43% and 59%, respectively.^7^ The results of a population study of young adults reported by Bartlett et al^11^ show that the prevalence of advanced erosive lesions (BEWE 2 and 3) for Eastern European countries was 17.7% (comparable to our own study), and was statistically significantly lower than in other countries, for example, in the UK it reached nearly 55%. It seems that the statistically significant differences observed in the presented studies are determined to some extent by the social and cultural context of the respective ethnolinguistic groups. Perhaps factors such as movements of the tongue, lips and facial muscles involved in the speaking process may further influence the formation of erosive tooth wear.^11^

In general, gender differences in the prevalence of ETW are well documented. The higher prevalence and severity of ETW in men than in women have been reported in many publications.^8,15,22,37^ In the Polish sample, conclusive results were obtained, although not for all the age groups studied. However, in the combined analysis of all three groups, it was found that gender was a risk factor for ETW, as it was more frequent in men.

It is commonly accepted that dietary and lifestyle changes in modern society affect oral health, not only with regard to caries, but also to non-carious lesions. Experts link high consumption of carbonated beverages in particular to the risk of developing erosive tooth wear.^19^ In Poland, the daily consumption of sweetened carbonated beverages averages 43 ml per person. According to data posted on the Eurostat portal, Poland ranks at about the average level of consumption of sweetened carbonated beverages as other European countries.^30^ Boys and young men consume them in the highest amounts. Among girls and young women, the consumption is moderate to relatively high, and this is a consistent characteristic in most of the European and world population.^23^ The data obtained clearly indicated that the maximum severity of erosive lesions correlated – especially in older groups, young adults and adults – with the total exposure to acidic drinks and foods. The most common dietary group correlating with ETW was sweetened carbonated beverages (e.g., cola and orangeade). Other authors have presented similar findings.^4,5,14,25,33,36,43^

Any self-report study carries the risk of obtaining falsified data. The comprehensive identification of risk factors in a clinical setting is based on a detailed dietary record conducted for several days, which is then analysed with the patient. Meanwhile, averaging the amount of acidic intake only gives an idea of the real situation. However, the results obtained speak in favor of the association between acidic diet and the prevalence of ETW in the study population. At the same time, it is worth mentioning that it was not uncommon for respondents to be convinced of the “safety” and low cariogenicity of consuming “lite” drinks.

Health status strongly correlated with level of education, as well as the socioeconomic status of the people surveyed. According to the study, education, level of knowledge and awareness play a more dominant role in personal health choices than do material factors.^12,16^ The questionnaire study showed interesting correlations between the prevalence of ETW and education and level of knowledge about erosive lesions. In the adult group, it was found that the amount of ETW was statistically significantly lower as the level of education increased. At the same time, the studied respondents’ level of knowledge relating to the effects of excessive consumption of acidic foods and drinks on teeth did not correlate with the prevalence and severity of ETW. Greater awareness does not always result in the elimination of bad habits, as seen in the present study. This points to the need to implement effective strategies to reduce the level of consumption of sweetened carbonated beverages. According to a study by Verploegen et al,^41^ patients obtain very little information from the dental team in a dentist’s office. More and more adults rely on obtaining health-related knowledge from the Internet.^40^ Moreover, the use of the Internet and social media can improve health awareness but not motivation, and it does not increase self-efficacy in health promotion. This indicates the importance of evidence-based information to change behaviour, particularly related to acidic beverage consumption among adolescents and young adults.

### Limitations of the Study

The study was carried out over a long period of time, so the different groups may have been affected by slightly different conditions. One can mention, for example, the rapid development of the market for carbonated beverages, energy drinks, flavoured waters, etc. The increase in the range of oral hygiene products recommended for people with ETW can be considered a positive development.

The need to standardise the questionnaire for statistical analysis resulted in the elimination of several questions in the presented study, for example, questions related to alcohol and psychotropic drug use as well as average income.

## CONCLUSION

The current state of knowledge clearly indicates that modern lifestyles promote erosive tooth wear, which will continue to occur in future patient populations. To date, there are few studies in the literature describing the prevalence of ETW in central and eastern Europe. The data that do exist indicate a lower prevalence and severity of ETW, which was confirmed by this study.

The analysis showed that, depending on age, multiple and significant risk factors for ETW become most apparent only in the 35-44 age group, especially with regard to general health. This suggests that the long-term impact of factors and their cumulative effects are critical to the development of ETW.

## Clinical relevance:

Present findings support the notion that erosive tooth wear (ETW) is less prevalent and severe in Poland compared to Western Europe, suggesting that there may be protective factors in the study population that warrant further investigation.
